# Novel Surgical Approach for Large Intraosseous Subchondral Cysts of Talus: A Case Report and Technical Innovation

**DOI:** 10.7759/cureus.52078

**Published:** 2024-01-11

**Authors:** Pradeep Moonot, Shubham Dakhode, Nikhil Karwande

**Affiliations:** 1 Orthopaedics, Mumbai Knee Foot Ankle Clinic, Mumbai, IND; 2 Orthopaedics, Sir H. N. Reliance Foundation Hospital, Mumbai, IND; 3 Orthopaedics, Breach Candy Hospital, Mumbai, IND

**Keywords:** bone grafting, novel surgical approach, intraosseous lesion, talar dome, subchondral cyst

## Abstract

Large subchondral bone cysts in the medial talar body and dome are common and can cause persistent pain and swelling during axial loading. Open debridement and bone grafting are often necessary to treat these lesions but can require extensive soft-tissue dissection or malleolar osteotomies. A 40-year-old woman presented with ankle pain and swelling for 1 year, worsening with activity and no history of trauma. X-rays showed a cystic lesion in the medial talar dome with no joint line disruption. CT confirmed the cystic lesion without bone collapse or expansion. An anterior approach to the ankle joint was extended to access the talar neck. A window was created in the talar neck to debride and curette the medial talar dome, and the void was filled with allograft. The patient was non-weight-bearing for 6 weeks, followed by gradual weight-bearing and ankle range of motion exercises starting on postoperative day 1. The patient returned to her pre-injury status within 3 months and was asymptomatic at the 6-year follow-up, with good bone graft integration and no symptoms. This technical note presents a novel approach to lesions of the medial talar body and dome through the talar neck, avoiding the need for malleolar osteotomy or disruption to the tibiotalar joint, and resulting in good functional outcomes.

## Introduction

Large subchondral bone cysts in the medial talar body and dome are relatively common and can also be associated with osteochondral lesions [1]. These lesions typically average around 1 cm in size, although larger than 2 cm is rare, and there have been reports of exceptionally large ganglions, up to 7 cm in size [2]. Patients experience intermittent pain, which often intensifies with activity. Axial loading can lead to persistent pain while walking due to joint irritation, and localized swelling may also be observed [3]. Radiographically, the lesion is characterized by a well-defined circular or oval radiolucent area, bordered by a rim of denser, sclerotic bone [4]. The standard approach for managing these lesions involves intralesional curettage followed by bone grafting, which is generally effective, with recurrence occurring in about 7% of cases [3]. In cases where the cyst is extensive and has caused significant damage to the talar dome, alternative options such as using fresh osteochondral allografts or vascularized bone grafts can be considered. If attempts to salvage the talus are unsuccessful, arthrodesis may be recommended [5-7]. Surgical access to these cysts usually involves a medial malleolar osteotomy in an open surgical procedure [8]. A unique case is presented in which a large intraosseous subchondral cyst of the talus, without joint involvement, was effectively treated using curettage and bone grafting. This was achieved through a novel approach via the talar neck, which did not require a medial malleolar osteotomy or disrupt the tibiotalar joint. The decision to operate on the patient was based on the symptomatic nature of the talus cyst and the potential for joint exposure under axial loading. This case report not only highlights the existence of a large intraosseous subchondral cyst of the talus but also introduces a novel approach to address such cysts.

## Case presentation

**Figure 1:**
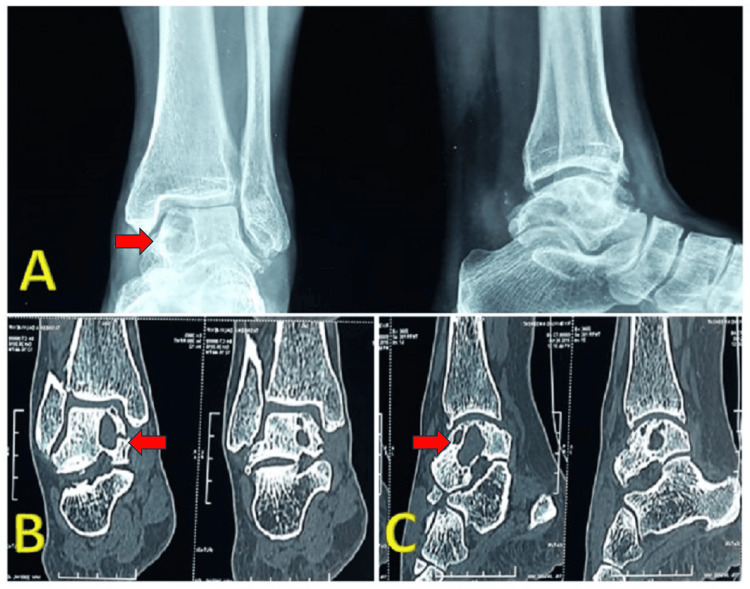
Preoperative Imaging Figure 1A shows preoperative x-ray showing radiolucent regions in the subchondral area of the medial talar dome and body of the talus, with no joint line disruption. Figure 2B (sagittal) and 2C (coronal) CT scan cuts showing radiolucent multiloculated cyst in the medial talar dome and body, without any signs of bone collapse or expansion.

A 40-year-old woman came to our clinic with persistent pain in her left ankle that had been ongoing for a year. The pain started as occasional but gradually became constant, making walking difficult and causing a noticeable limp. Swelling around the ankle was evident and tended to worsen during activities. The patient denied any history of trauma. During the physical examination, the ankle had a normal range of motion, but passive dorsal hyperflexion and inversion caused pain. A mild joint effusion was observed along the medial and lateral joint lines, and palpation over the anteromedial joint line of the ankle elicited deep-seated pain. Neurological and vascular assessments yielded normal findings. Radiographs showed a radiolucent region in the subchondral area of the medial talar dome and body of the talus, with no indication of joint line disruption. Further evaluation through computed tomography revealed a radiolucent multiloculated cyst in the medial talar dome and body, without any signs of bone collapse or expansion (Figure 1).

Due to the symptomatic nature of the cyst, surgical intervention was recommended for the patient.

Surgical technique

**Figure 2:**
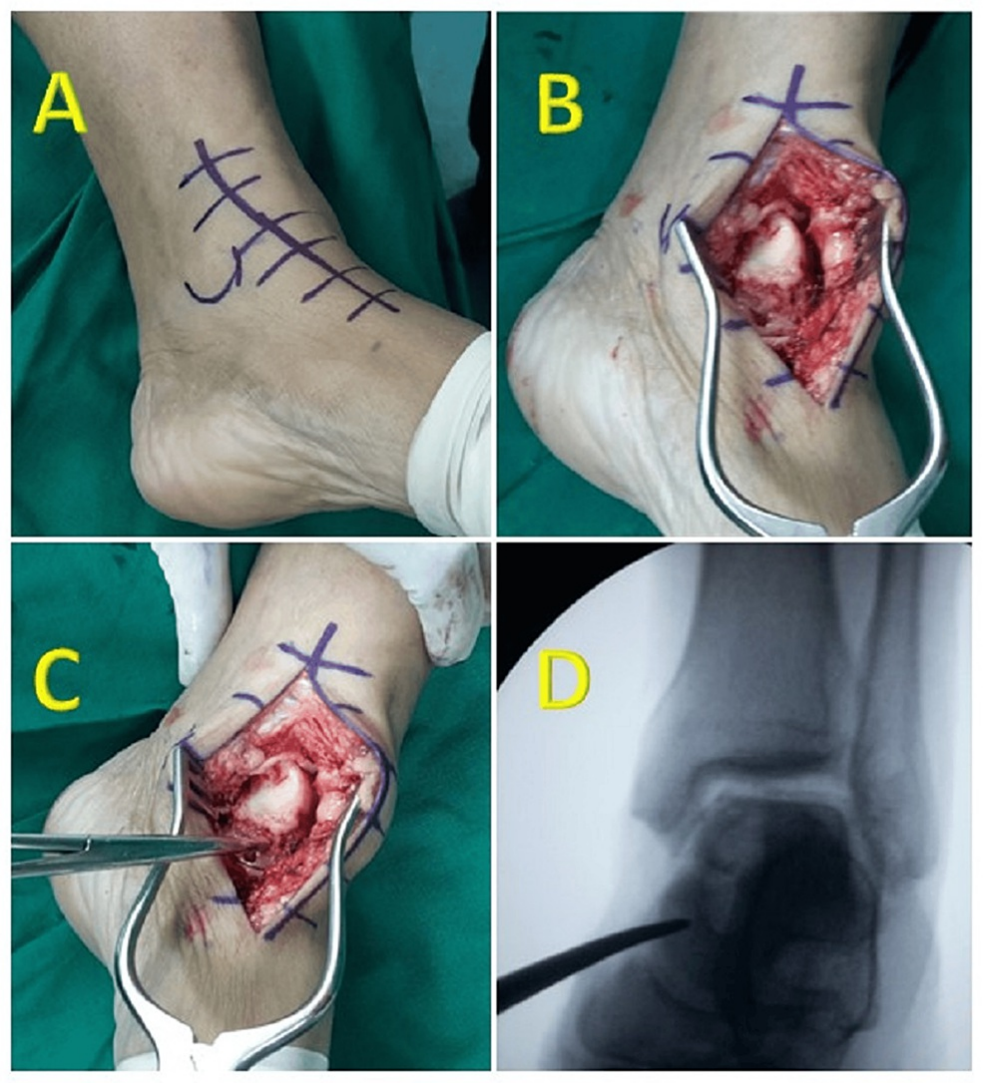
Sequential Surgical Steps Figure 2A shows incision from the medial malleolus to the talonavicular joint to approach medial talar neck. Figure 2B shows a final exposure of talar neck. Figure 2C shows identification of medial talar neck. Figure 2D shows confirmation of talar neck under fluoroscopy.

The surgical exploration involved making a curved 2-cm incision from the medial malleolus to the talonavicular joint. The skin and fascia were cut to expose the underlying medial talar neck, and fluoroscopy was used to confirm its location (Figure 2).

**Figure 3:**
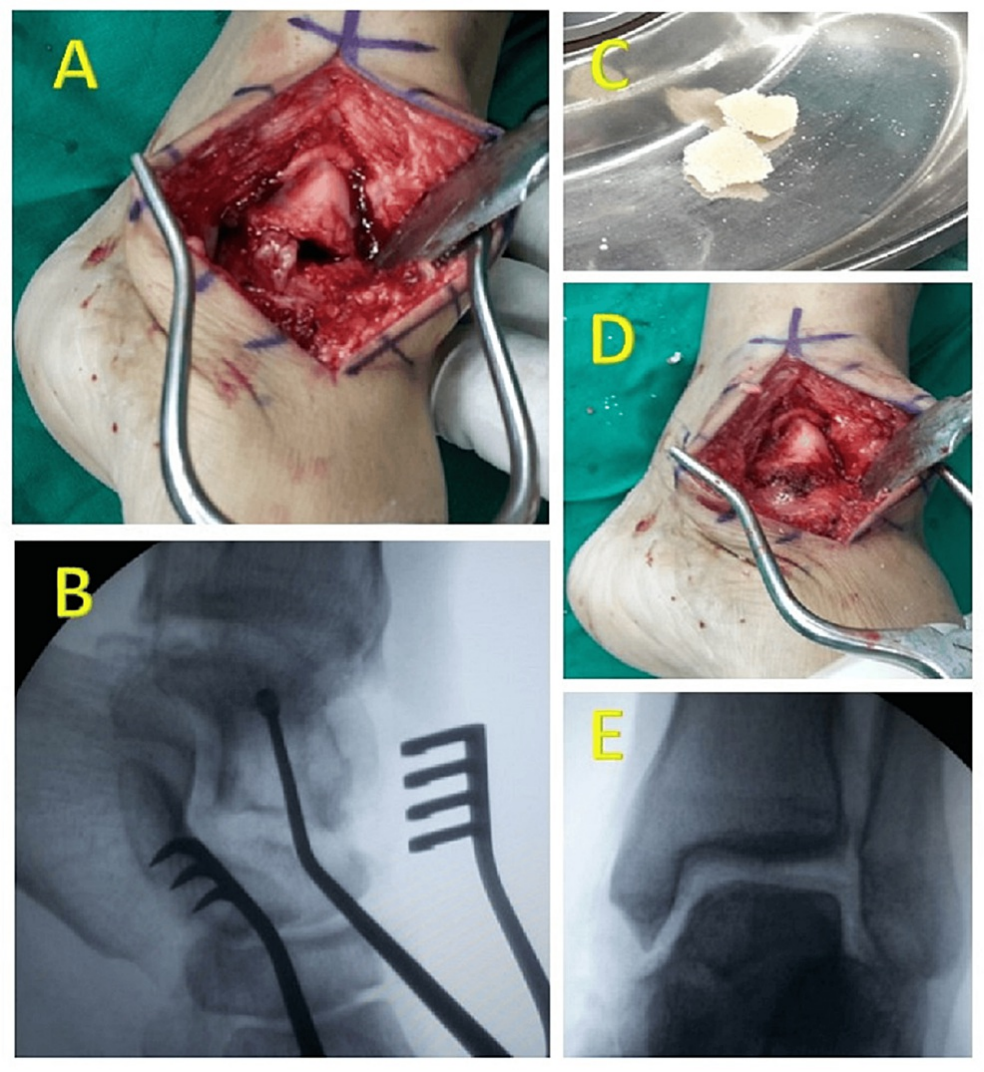
Sequential Surgical Steps Figure 3A shows a carefully made window in the medial talar neck. Figure 3B shows an angled curette for the thorough removal of the cyst. Figure 3C shows the allograft used. Figure 3D shows the complete packing of the cyst with bone graft. Figure 3E shows postoperative fluoroscopic image showing the cyst completely filled with bone graft.

A window was carefully made in the medial talar neck and enlarged with appropriately sized curettes to access the medial talar dome. The cyst was meticulously removed using curettage, with the final stage involving precise placement of an angled curette to ensure thorough removal of the cyst, and the resulting cavity was filled with allograft (Figure 3).

**Figure 4:**
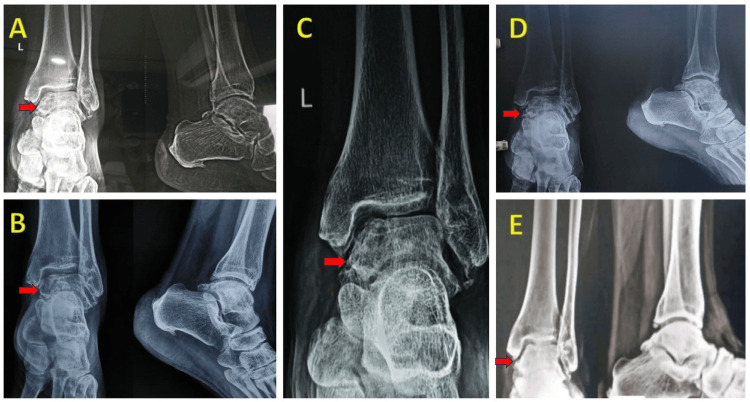
Sequential Radiographs Serial yearly follow-up x-rays showing good integration of bone graft with some signs of degenerative changes and no recurrence. Figure 4A - 2017, Figure 4B - 2018, Figure 4C - 2019, Figure 4D - 2021, Figure 4E - 2022.

The patient was immobilized in a plaster cast for two weeks, followed by ankle range of motion exercises and toe-touch weight bearing after four weeks. Full weight-bearing was permitted after six weeks, and by the eighth week, the patient had resumed regular activities with a full range of motion and no pain. No complications occurred during or after the surgery, and regular follow-up examinations showed excellent outcomes. Radiographs revealed satisfactory integration of the bone graft, with some signs of degenerative changes in the tibiotalar joints (Figure 4), but the patient remained asymptomatic. There was no recurrence of the condition at the latest follow-up, spanning six years.

## Discussion

Large subchondral cysts in the talus present challenges in treatment, and surgical intervention may not consistently produce satisfactory outcomes. These cysts can lead to osteochondral lesions due to inadequate subchondral support, potentially causing arthritis. Additionally, these lesions have poor healing due to nonviable subchondral bone and a scarcity of mesenchymal stem cells for lesion restoration [9,10]. Various approaches have been explored for treating osteochondral lesions with subchondral cysts. Kolker et al [9] performed open antegrade autologous bone grafting in 13 patients with talar lesions, reporting good to fair outcomes in seven patients and clinical failure in six. However, they do not recommend this procedure as a standalone primary treatment for advanced lesions with deficient or absent overlying cartilage. Tanaka et al [6] used a vascularized medial calcaneal bone graft for large medial talar osteochondral lesions in four patients, achieving fair to good outcomes. However, their technique relies on residual cartilage.

It's important to note that these studies primarily address osteochondral lesions and not isolated subchondral cysts. In our case, the patient had a solitary subchondral cyst without an associated osteochondral lesion. Additionally, the medial malleolar osteotomy, which was required in the above studies to access the lesion, carries its own complications, such as malunion, nonunion, exposure of the tibial articular surface, and secondary arthritis due to osteotomy site incongruence [11]. However, our approach, using a talar neck window, avoided the need for malleolar osteotomy. Autologous bone grafting, a common orthopedic procedure, is not without complications. A systematic review by Dimitriou et al [12] identifies donor site morbidity, including chronic pain, infection, and neurovascular disturbances, hindering postoperative rehabilitation during the initial recovery phase. In our case, we opted for allograft use to fill the void, mitigating complications associated with autografts. Consequently, our technique may be considered less invasive and safer, although it is not without potential risks and complications, such as talar neck fracture and avascular necrosis of the talus, which, fortunately, were not encountered in our experience.

This case report illustrates a successful and innovative approach to managing a large intraosseous subchondral cyst of the talus. The use of a talar neck window avoided the need for medial malleolar osteotomy or tibiotalar joint disruption, successfully addressing the symptomatic cyst while preserving joint integrity. The patient's rapid recovery, excellent clinical outcome, and absence of recurrence over a 6-year follow-up period highlight the feasibility and efficacy of this technique. The talar neck approach also offers advantages over the traditional medial malleolar osteotomy approach, as it avoids the morbidity associated with a medial malleolar osteotomy and provides direct access to the medial talar dome and body without disrupting the tibiotalar joint. It is important to note that the decision to use this approach should be made on a case-by-case basis, taking into account the characteristics of the cyst. While further research and larger case series are needed to validate its broader applicability, this novel approach offers a promising alternative for the treatment of talar cysts and underscores the importance of innovation in orthopedic surgery to optimize patient outcomes.

## Conclusions

This case report presents a unique instance in which a large intraosseous subchondral cyst of the talus, without joint involvement, was successfully treated using curettage and bone grafting. The novel approach via the talar neck was used, eliminating the need for a medial malleolar osteotomy or disruption of the tibiotalar joint. This report not only highlights the existence of large intraosseous subchondral cysts of the talus but also introduces a new approach to addressing such cysts.

## References

[1] Sharma S, Gupta P, Sharma S, Singh M, Singh D (2012). Primary aneurysmal bone cyst of talus. J Res Med Sci.

[2] Pope TL Jr, Fechner RE, Keats TE (1989). Intra-osseous ganglion. Report of four cases and review of the literature. Skeletal Radiol.

[3] Nigrisoli P, Beltrami P (1971). Subchondral cysts of bone. Lo Scapello.

[4] Wise DI (1992). Intraosseous ganglia: radioisotope bone imaging. J R Coll Surg Edinb.

[5] Koulalis D, Schultz W (2000). Massive intraosseous ganglion of the talus: reconstruction of the articular surface of the ankle joint. Arthroscopy.

[6] Tanaka Y, Omokawa S, Fujii T, Kumai T, Sugimoto K, Takakura Y (2006). Vascularized bone graft from the medial calcaneus for treatment of large osteochondral lesions of the medial talus. Foot Ankle Int.

[7] Raikin SM (2009). Fresh osteochondral allografts for large-volume cystic osteochondral defects of the talus. J Bone Joint Surg Am.

[8] Jamshidi K, Kargar Shooroki K, Sharifi Dalooei SM, Mirzaei A (2023). Intraosseous ganglion cyst of the talus treated with curettage and bone grafting through a medial malleolus osteotomy. Foot Ankle Int.

[9] Kolker D, Murray M, Wilson M (2004). Osteochondral defects of the talus treated with autologous bone grafting. J Bone Joint Surg Br.

[10] Robinson DE, Winson IG, Harries WJ, Kelly AJ (2003). Arthroscopic treatment of osteochondral lesions of the talus. J Bone Joint Surg Br.

[11] Angermann P, Jensen P (1989). Osteochondritis dissecans of the talus: long-term results of surgical treatment. Foot Ankle.

[12] Dimitriou R, Mataliotakis GI, Angoules AG, Kanakaris NK, Giannoudis PV (2011). Complications following autologous bone graft harvesting from the iliac crest and using the RIA: a systematic review. Injury.

